# Engineering cellulases for conversion of lignocellulosic biomass

**DOI:** 10.1093/protein/gzad002

**Published:** 2023-03-24

**Authors:** Yogesh B Chaudhari, Anikó Várnai, Morten Sørlie, Svein J Horn, Vincent G H Eijsink

**Affiliations:** Faculty of Chemistry, Biotechnology, and Food Science, NMBU-Norwegian University of Life Sciences, P.O. Box 5003, 1432 Ås, Norway; Faculty of Chemistry, Biotechnology, and Food Science, NMBU-Norwegian University of Life Sciences, P.O. Box 5003, 1432 Ås, Norway; Faculty of Chemistry, Biotechnology, and Food Science, NMBU-Norwegian University of Life Sciences, P.O. Box 5003, 1432 Ås, Norway; Faculty of Chemistry, Biotechnology, and Food Science, NMBU-Norwegian University of Life Sciences, P.O. Box 5003, 1432 Ås, Norway; Faculty of Chemistry, Biotechnology, and Food Science, NMBU-Norwegian University of Life Sciences, P.O. Box 5003, 1432 Ås, Norway

**Keywords:** biorefining, cellulase, lignocellulose, processivity, thermostability

## Abstract

Lignocellulosic biomass is a renewable source of energy, chemicals and materials. Many applications of this resource require the depolymerization of one or more of its polymeric constituents. Efficient enzymatic depolymerization of cellulose to glucose by cellulases and accessory enzymes such as lytic polysaccharide monooxygenases is a prerequisite for economically viable exploitation of this biomass. Microbes produce a remarkably diverse range of cellulases, which consist of glycoside hydrolase (GH) catalytic domains and, although not in all cases, substrate-binding carbohydrate-binding modules (CBMs). As enzymes are a considerable cost factor, there is great interest in finding or engineering improved and robust cellulases, with higher activity and stability, easy expression, and minimal product inhibition. This review addresses relevant engineering targets for cellulases, discusses a few notable cellulase engineering studies of the past decades and provides an overview of recent work in the field.

## Graphical abstract

**Figure:**
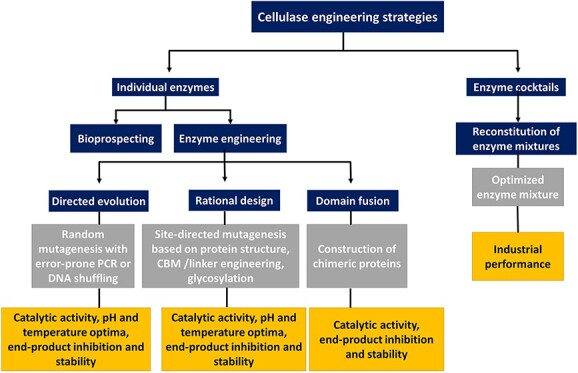

## Introduction

With increasing concerns about global sustainability, the transition from today’s fossil-based economy toward a more sustainable biobased economy has received significant interest worldwide (Menon and Rao, 2012). Lignocellulosic biomass represents the Earth’s largest reservoir of renewable resources, with an estimated 180 billion tons of production annually (Paul and Dutta, 2018). Because of its availability, wide distribution, renewability, low cost, as well as current underutilization, lignocellulosic biomass is considered as a promising raw material for producing biofuels and value-added products (Wyman and Goodman, 1993, Mabee *et al.*, 2011).

Lignocellulosic biomass, such as wood or corn stover, is mainly composed of three main components, namely cellulose (30–50%, w/w), hemicellulose (20–40%, w/w) and lignin (15–25%, w/w) (Chen, 2014, Champreda *et al.*, 2019). Cellulose, a linear homopolymer of D-glucose linked by β-(1 → 4)-glycosidic bonds, represents the major component of the plant cell wall (30–50%, w/w, of the total dry matter). The cellulose chains tend to be organized in highly ordered crystalline structures, in which the polysaccharides are held together by a dense network of hydrogen bonds. In the plant cell wall, cellulose microfibrils are embedded in a network of hemicelluloses and lignin. Hemicelluloses comprise a diverse group of branched polysaccharides that consist, to various extents, of pentoses (D-xylose and L-arabinose), hexoses (D-mannose, D-glucose and D-galactose) and sugar acids. They may be acylated to various degrees with acetyl, feruloyl and/or *p*-coumaroyl groups. The most abundant hemicellulose types are glucuronoxylans, glucomannans and xyloglucans. A fraction of these hemicelluloses, often called recalcitrant hemicellulose, directly adheres to and coats cellulose microfibrils. The remaining hemicellulose may be interlinked through ester and ether bonds and forms an intricate three-dimensional network around the cellulose skeleton (Scheller and Ulvskov, 2010). Lignin is a heteropolymer of phenylpropanoid subunits (guaiacylpropane, syringylpropane and *p*-hydroxyphenylpropane) that are covalently coupled primarily through ether and carbon–carbon linkages. In addition, lignocellulosic biomass may contain minor amounts of pectin, proteins, lipids and minerals. The complex, inhomogeneous and dense arrangement of all the non-cellulose components creates a physical barrier around the cellulose skeleton. This arrangement, together with the crystalline nature of the cellulose itself, makes plant biomass resilient to biochemical degradation. Plant biomass recalcitrance has been identified as a significant hindrance to lignocellulose depolymerization in nature and in biorefining industries.

Enzymatic depolymerization of cellulose to glucose provides an alternative for chemical depolymerization methods and is considered a crucial step in the environmentally sustainable conversion of plant biomass to sugars that can be fermented to value-added products, such as fuels, chemicals and foods (Zhu *et al.*, 2016). Such depolymerization requires the pretreatment of the biomass to remove and/or remodel other plant cell wall components and to reduce cellulose crystallinity. Of note, enzymes used for depolymerization of cellulose and other components in the plant biomass may also be used to produce and refine cellulose-based novel materials, such as cellulose nanofibers (de Aguiar *et al.*, 2020, Yang *et al.*, 2020). However, protein engineering studies aimed at tailoring cellulases for this particular purpose are rare (e.g. Rahikainen *et al.*, 2019).

**Fig. 1:**
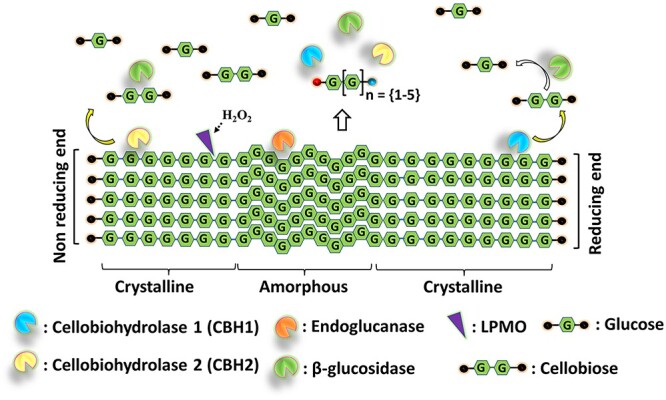
Degradation of cellulose by the synergistic action of cellulases (cellobiohydrolases, endoglucanases), β-glucosidases and LPMOs. The concomitant action of cellobiohydrolases (CBHs), endoglucanases (EGs) and LPMOs releases a mixture of native and oxidized cello-oligosaccharides. Soluble products released by the LPMOs will mostly be 2–6 sugars long and will be oxidized at one of the chain ends, as indicated by the red and blue dots. The products of cellulases and LPMOs are converted by β-glucosidases (BGLs) to monomers as the final product of cellulose saccharification.

Enzymatic hydrolysis of cellulose is challenging, even for pure cellulose, and requires the synergistic action of three types of hydrolytic enzymes: cellobiohydrolases (CBHs), endoglucanases (EGs) and β-glucosidases (BGLs) (Payne *et al.*, 2015). As depicted in Fig. 1, EGs (EC 3.2.1.4) cleave internal β-(1 → 4)-glycosidic bonds randomly and are thought to act in the more amorphous regions of cellulose where they will generate new chain ends for CBHs (Lynd *et al.*, 2002, Sweeney and Xu, 2012). CBHs are processive cellulases that act on the non-reducing (EC 3.2.1.91) or reducing (EC 3.2.1.176) ends of cellulose chains releasing disaccharides (i.e. cellobiose). Of note, several CBHs are known to be capable of initial endo-binding, next to attacking chain ends (Ståhlberg *et al.*, 1993, Kurašin and Väljamäe, 2011). Solubilized cellobiose and cello-oligosaccharides are converted to glucoses by BGLs (EC.3.2.1.21) that act from the non-reducing end (Fig. 1).

In 2005, Vaaje-Kolstad et al. described a new type of protein that significantly boosts the hydrolyzing ability of hydrolases that act on crystalline polysaccharides (Vaaje-Kolstad *et al.*, 2005). In 2010 and 2011 (Vaaje-Kolstad *et al.*, 2010, Forsberg *et al.*, 2011), it was shown that these proteins, which are now called lytic polysaccharide monooxygenases (LPMOs; EC 1.14.99.54), enable aerobic microbes to cleave glycosidic bonds in the crystalline parts of cellulose through oxidation of the reducing (C_1_ carbon; EC 1.14.99.54) or non-reducing chain end (C_4_ carbon; EC 1.14.99.56) at the scissile bond, generating a D-gluconate or a 4-keto-D-glucose, respectively (Horn *et al.*, 2012, Vu *et al.*, 2014). Thus, LPMOs add an ‘endo’-type of activity to the enzyme mix that classical EGs likely cannot provide (Fig. 1), and their discovery has contributed to significant improvements in the efficiency of commercial cellulase cocktails (Costa *et al.*, 2020).

Microorganisms, in particular plant biomass-decomposing soil bacteria and fungi, are the most important source of enzymes for plant biomass depolymerization (Himmel *et al.*, 2010, Koeck *et al.*, 2014). Filamentous fungi secrete multiple, often multi-domain, cellulases, whereas many anaerobic bacteria, as well as few anaerobic fungi, produce a complex of cellulolytic enzymes associated in a structure referred to as the cellulosome (Bayer *et al.*, 2004). In some anaerobic bacteria, individual cellulases are displayed on the microbial surface or are released in extracellular vesicles (Arntzen *et al.*, 2017, La Rosa *et al.*, 2022). Although both bacteria and fungi can produce cellulases, fungi are considered better candidates for cellulase production due to the efficiency of their enzymes and their capacity to produce large amounts of extracellular enzymes (Mondal *et al.*, 2019).

There are multiple reviews on cellulases and cellulase engineering. Fungal cellulases and their engineering have been reviewed by Payne *et al.* (2015) in an impressively comprehensive review that addresses many (general) aspects of cellulase structure and function in much detail. Other useful reviews focusing on engineering cellulases include (Bommarius *et al.*, 2014, Greene *et al.*, 2015, Contreras *et al.*, 2020a, Zhang *et al.*, 2021). In this short review, we discuss engineering strategies used for increasing the industrial performance of cellulases, presenting a few selected highlights from the past and recent advancements. After a summary of sequence-based cellulase families, we discuss cellulase properties that are considered industrially relevant and that may be the target of protein engineering endeavors. The industrial application of cellulases requires efficient production strains capable of producing optimized enzyme blends at low cost as well as deep insight into the interplay between the various enzymes in such blends. Although not the main focus of this review, these crucial aspects are also shortly discussed.

### Cellulases

Cellulose-depolymerizing enzymes, as all enzymes, can be described by (and, consequently, classified based on) their amino acid sequence, three-dimensional fold and catalytic mechanism. Cellulases belong to the large class of glycoside hydrolases (GHs) in the manually curated Carbohydrate-Active enZymes database (CAZy) (http://www.cazy.org/) (Drula *et al.*, 2022), which classifies carbohydrate-active enzymes into families of structurally similar proteins. GHs are the primary drivers of enzymatic polysaccharide degradation in nature and comprise a vast collection of enzymes.

**Fig. 2:**
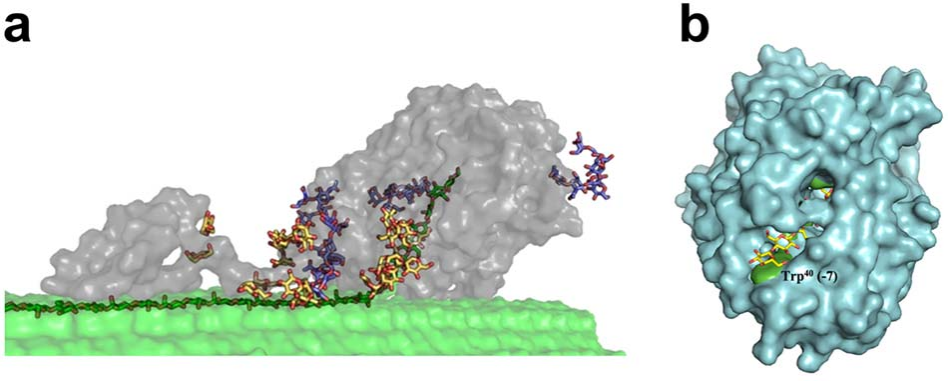
Structure and cellulose binding of a GH family 7 cellobiohydrolase. (**a**) Structure of the CBH *Tr*Cel7A from *T. reesei*, comprised of a catalytic domain (CD) (right grey part), a CBM (left grey part), and a connecting linker domain, bound to cellulose (Greene *et al.*, 2015). The enzyme acts on a single cellulose chain that needs to be extracted from its crystalline context (dark green carbons) and guided into the catalytic tunnel. The structures shown with yellow and blue carbons are *O*-glycans on the linker and CBM, and *N*-glycans on the catalytic domain, respectively. This picture was taken from Taylor *et al.* (2018). (**b**) Structure of the catalytic domain of *Tr*Cel7A bound to cellononaose (PDB code 4C4C; the sugar molecule has yellow carbons) (Knott *et al.*, 2014). The picture provides a view on the substrate-binding tunnel that contains four conserved tryptophan residues (Trp38, Trp40, Trp367 and Trp376; green) that are stacking with the cellulose substrate, as shown in Fig. 3. Trp40, at what has been called the tunnel ‘entrance’, corresponding to the −7 subsite, is labeled.

CBHs are mainly found in GH families 6, 7, 9 and 48. The CBHs found in fungi generally belong to families GH6 and GH7, while bacterial CBHs are found in families GH6, GH9 and GH48 (Payne *et al.*, 2015, CAZypedia, 2018, Drula *et al.*, 2022). CBHs are processive enzymes whose catalytic site often has a tunnel-like topology (Fig. 2). This topology facilitates a catalytic process whereby a single cellulose chain slides along the catalytic center while cellobiose units are being released, starting either at the reducing end (by enzymes referred to as CBH I and occurring in families GH7 and GH48) or the non-reducing end (by enzymes referred to as CBH II and occurring in families GH6 and GH9) (Beckham *et al.*, 2014). The main cellulolytic enzymes produced by filamentous fungi known for strong cellulolytic activity, such as *Trichoderma reesei*, are family GH7 CBHs, and these enzymes are particularly important in industrial fungal cellulase cocktails (Payne *et al.*, 2015). Therefore, these CBHs have been primary targets for cellulase engineering (Taylor *et al.*, 2018). One of the most studied CBHs is *Tr*Cel7A from *T. reesei*, a multi-modular enzyme with a GH7 catalytic domain (CD; Divne *et al.*, 1994) that is connected to a Family 1 carbohydrate-binding module (CBM) by a flexible linker (Fig. 2). Interestingly, adding to the spectrum of possible engineering targets, this enzyme is heavily glycosylated (Fig. 2) and recent work has shown that these glycosylations may make specific contributions to cellulase efficiency by interacting with the substrate (Amore *et al.*, 2017).

EGs, are found in many GHs families, including GH5, GH6, GH7, GH8, GH9, GH10, GH12, GH26, GH44, GH45, GH48, GH51, GH74, GH124, GH131 and GH148 (Drula *et al.*, 2022), with members of family GH5 and a well-known fungal GH7 EG, *Tr*Cel7B being among the most studied. The active sites of EGs form an open cleft that can accommodate a single cellulose chain in an amorphous region and cleave it randomly along the chain (Davies and Henrissat, 1995, Lynd *et al.*, 2002, Sidar *et al.*, 2020). EGs can occur as single-domain or multi-modular enzymes. One of the major EGs produced by *T. reesei*, *Tr*Cel7B, accounting for 10–15% of secreted proteins during growth on cellulose, is composed of a GH7 domain and a CBM1 (Kleywegt *et al.*, 1997). Importantly, although CBHs seem tailored to act on recalcitrant forms of cellulose, enabled by their processive nature (Horn *et al.*, 2006, Beckham *et al.*, 2014) (Fig. 2), EGs are not necessarily good and true cellulases. For example, the GH5 family includes enzymes that show minute activities on cellulose, while being efficient in degrading less recalcitrant β-(1 → 4) glycans such as β-glucan and glucomannan. Thus, for judging the potential of described enzymes or the success of an engineering effort, it is crucial to consider which substrates were used for enzyme characterization.

BGLs occur in families GH1, GH3, GH9 and GH30. In general, BGLs have a catalytic pocket (subsite −1), which accommodates the nonreducing-end glucose of the cello-oligosaccharide substrate. BGLs are formally not cellulases but are crucial in cellulolytic enzyme cocktails not only to reach the end product, glucose, but also to alleviate the inhibition of cellulases by their oligomeric products.

## Protein engineering of cellulolytic enzymes

Depending on their application, cellulases are either used individually (e.g. paper manufacturing, cellulose fiber upgrading or fruit juice extraction) or in a cocktail (e.g. biofuel industry, animal feed production and detergent industry). Accordingly, cellulases may be engineered for optimizing their activity individually or in combination with other cellulolytic enzymes. Even with the focus being on improved processing of lignocellulosic biomass, academic work on cellulase engineering generally entails a one-enzyme-at-the-time approach. There are numerous engineering targets for cellulases, some common to all enzymes, and some addressing specific challenges related to the processing and valorization of lignocellulosic biomass. As to the latter, enzyme costs are a major overall cost driver and enzymes are needed in amounts so large that lignocellulose biorefineries may need to include an on-site enzyme production facility. Clearly, there is a lot to gain from enzymes that are easier to produce, more active and more stable. Natural enzymes will usually not do the job, since process conditions (such as operating temperature and pH, and end-product concentration) often differ from the conditions in native environments. In addition, natural enzymes may not be sufficiently active, even at optimal conditions, to allow for economically viable bioprocesses.

In short, engineering targets for cellulases and the rationale behind are as follows:

- Improved catalytic activity, including, e.g. an optimized pH-activity profile; this is a general strategy that may give lower enzyme consumption and increased process efficiency.
- Improved thermal stability at relevant pH; this is a general strategy for lower enzyme consumption that also may allow running reactions at increased temperatures, which has several advantages, including reduced risks for microbial contamination.
- Reduced product inhibition and changed enzyme processivity. Product inhibition may be a problem at industrial high-concentration conditions and relates to the enzyme’s affinity for glucose and short cello-oligomers. This affinity also affects the degree of enzyme processivity, which is a cellulase property that receives much attention because it is needed for efficiency on the more recalcitrant (crystalline) parts of cellulose while at the same time making enzymes slow, due to slow product release (Horn *et al.*, 2006, Vuong and Wilson, 2009, Beckham *et al.*, 2014, Kuusk *et al.*, 2015, Vermaas *et al.*, 2019, Olsen *et al.*, 2020, Sørlie *et al.*, 2020). Of note, many engineering efforts made to increase the activity of CBHs deal with manipulating substrate affinity, addressing the delicate balance between having affinity that is high enough to access the substrate and low enough to allow for efficient substrate and product release (see below for examples).
- Adding or removing CBMs. This may increase enzyme efficiency although the expected increased substrate affinity may be a two-edged sword in high dry-matter processes (Várnai *et al.*, 2013). Positive effects of CBMs on enzyme stability have also been described (Sidar *et al.*, 2020).
- Changing the linkers that connect CBMs and catalytic domains. Nature employs many different linkers with varying degrees of flexibility, length and glycosylation and, although rational engineering of linker sequences seems not yet possible, it is clear that functional variation can be obtained by changing linkers (Payne *et al.*, 2013, Papaleo *et al.*, 2016, Amore *et al.*, 2017, Sørensen and Kjaergaard, 2019).
- Changing glycosylation. As alluded to above, glycosylation may affect the efficiency of certain cellulases. Glycosylation can be engineered by removing or introducing glycosylation sites, by changing linkers (see above), and, in principle, also by changing the glycosylation machinery of the enzyme-producing microbe.
- Reducing enzyme adsorption to lignin. The loss of cellulase activity due to enzyme adsorption to the lignin present in pretreated lignocellulosic biomass is generally considered a problem, that perhaps is related to the presence of CBMs (Kumar *et al.*, 2012, Rahikainen *et al.*, 2013, Kellock *et al.*, 2017).
- Improved activity and stability in non-conventional media. This is relevant when using cellulases for refining of cellulose fibers for material applications (Ribeiro *et al.*, 2019).

The methodologies used for cellulase engineering are no different from those used for other enzymes, including directed evolution-type approaches (Heinzelman *et al.*, 2009, Bornscheuer *et al.*, 2019), rational mutagenesis and all sorts of hybrid forms where (semi-)rational strategies are used to reduce the library sizes in screening-based efforts. Rational engineering is increasingly supported by successful computational methods including artificial intelligence (Wijma *et al.*, 2014, Khersonsky *et al.*, 2018, Gado *et al.*, 2021, Lu *et al.*, 2022).

Most importantly, screening of cellulase properties that are industrially relevant requires the use of true substrates during screening. Such true substrates, like steam-pretreated corn stover, are insoluble and heterogeneous, which complicates both handling and product analysis. The use of such substrates may be particularly complicated in high-throughput screening-based approaches, where typically thousands of enzyme variants need to be assessed using automated pipetting, although microtiter plate-based screening methods have been described (e.g. Chundawat *et al.*, 2008). Using realistic substrates will be easier when using lower-throughput approaches, for example, for screening a limited set of rationally designed mutants. For practical purposes, cellulase engineering studies may employ soluble cellulose variants such as carboxymethyl cellulose, or even short soluble chromogenic cello-oligomers. While studies using such substrates may generate interesting results, their relevance for increasing the efficiency of industrial lignocellulose processing may be limited.

Because of their superior efficiency and industrial use, fungal cellulases were the targets of most published cellulase engineering studies. For the period before 2015, such studies are extensively reviewed in Payne *et al.* (2015). Recent reviews with more or less comprehensive overviews of more recent cellulase engineering studies date from 2020 (Contreras *et al.*, 2020a) and 2021 (Zhang *et al.*, 2021). These include tables that list, and shortly describe, individual studies published, primarily, in the period of 2010–2020. In the sections below, we provide a brief overview of relatively recent engineering efforts and achievements. Additionally, a more comprehensive summary of recent engineering efforts can be found in Supplementary Table 1.

### Engineering for improved enzyme activity

#### Cellobiohydrolases (CBHs)

**Fig. 3:**
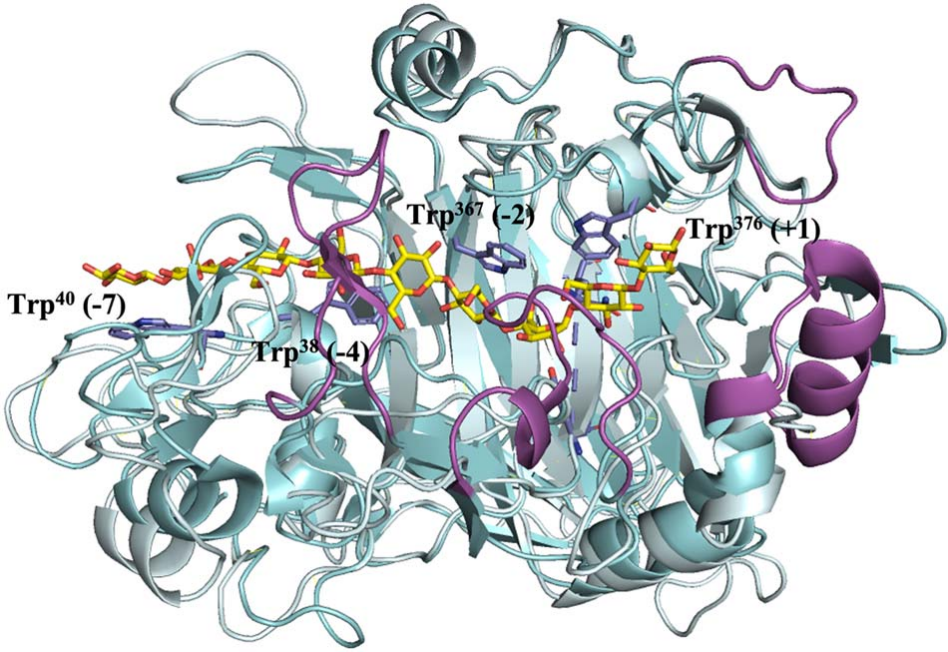
Superimposed crystal structures of the catalytic domains of cellobiohydrolase *Tr*Cel7A (cyan, PDB code 4C4C) bound to cellononaose (Knott *et al.*, 2014) and the endoglucanase *Tr*Cel7B (pale cyan to underscore similarities, PDB code 1EG1) (Kleywegt *et al.*, 1997). The figure shows that the catalytic domains of the enzymes are similar, but that *Tr*Cel7A has additional loops (highlighted in magenta) that create a tunnel covering the substrate-binding cleft. The substrate (yellow carbons) covers subsites +2 to −7 and interacts, in *Tr*Cel7A, with four tryptophans (side chains shown with blue carbons), Trp38 (−4 subsite) Trp40 (−7 subsite), Trp367 (−2 subsite) and Trp376 (+1 subsite). Three of these tryptophans are conserved in *Tr*Cel7B. The substrate is cleaved between the sugars bound to subsites −1 and + 1. The loops that shape the substrate-binding clefts and tunnels of GH7 cellulases have been the subject of many engineering studies. For example, Schiano-di-Cola *et al.* (2019) have undertaken a detailed study addressing the impact of such loops on the kinetics of substrate hydrolysis and substrate binding in *Tr*Cel7A.

Characterization of a growing number of CBHs has shed light on their detailed mechanism and led to the identification of key amino acid residues important for substrate binding, processivity and product dissociation (Payne *et al.*, 2015). Depending on the substrate, there is a trade-off between stronger substrate/product binding and high processivity vs. weaker substrate binding, less processivity and faster substrate/product dissociation. Strong substrate binding brings a risk of formation of non-productive enzyme–substrate complexes in which a cellulase is strongly, and more or less permanently, bound to the substrate without being able to cleave it (Horn *et al.*, 2006, Igarashi *et al.*, 2011, Kurašin and Väljamäe, 2011, Vermaas *et al.*, 2019). Thus, rather than focusing on the reactivity of the catalytic center itself, CBH engineering has focused on manipulating substrate and product binding, either by changing residues that interact with the substrate and/or by engineering loops that shape the catalytic cleft/and tunnel and may affect the ease at which substrates are bound and products are released (Von Ossowski *et al.*, 2003, Payne *et al.*, 2015) (Fig. 3). Individually mutated residues include the aromatic residues that typically line the substrate-binding clefts of processive cellulases (and chitinases), likely facilitating the ‘sliding’ of the substrate through the catalytic cleft or tunnel (Varrot *et al.*, 2003) (Fig. 3).

Reported engineering efforts with CBHs often concern manipulation of the structure, length and/or flexibility of substrate-enclosing loops, e.g. by engineering disulfide bridges or by changing loop length or sequence composition (Voutilainen *et al.*, 2010, Sørensen *et al.*, 2017, Taylor *et al.*, 2018, Schiano-di-Cola *et al.*, 2019). Other engineering targets include specific substrate-binding residues at the entrance of the catalytic tunnel, and/or inside the catalytic tunnel (Nakamura *et al.*, 2013, Kari *et al.*, 2014). These approaches have shed light on structure–function relationships in CBHs (e.g. Nakamura *et al.*, 2013, Schiano-di-Cola *et al.*, 2019), and have yielded enzymes with improved properties. For example, Voutilainen *et al.* (2010) obtained thermostable variants of Cel7A from *Talaromyces emersonii* by introducing disulfide bridges in loops that form, or are close to, the substrate-binding tunnel, and these mutants showed improved activity on Avicel at higher temperatures. Kari *et al.* (2014) showed that weakening substrate affinity by mutating Trp-38 in the −4 subsite of *Tr*Cel7A (Fig. 3) lowers substrate affinity but increases the activity on Avicel 2-fold. Of note, mutation of Trp-40 at the tunnel entrance (−7 subsite, Fig. 3) reduced activity toward crystalline substrates (Nakamura *et al.*, 2013).

Much work on GH7 CBHs has addressed the roles of substrate-enclosing loops near the substrate entrance, in particular near the −4 subsite (Sørensen *et al.*, 2017, Taylor *et al.*, 2018, Schiano-di-Cola *et al.*, 2019). Sørensen *et al.* (2017) showed that mutations in a loop covering subsite −4 (the B2 loop) reduced substrate affinity and increased activity on crystalline cellulose (Avicel) two-fold (not unlike the mutation of Trp-38, discussed above). Inspired by comparisons with endo-acting *Tr*Cel7B, Schiano-di-Cola *et al*. (2019) mutated tunnel-forming loops in *Tr*Cel7A and found that deletions in the B2 loop covering the −4 subsite had the largest effects. In contrast to the site-directed mutations described by Sørensen *et al.* (2017), the loop deletions reduced activity on crystalline cellulose, leading to the conclusion that the B2 loop is a key determinant of *Tr*Cel7A’s function as a CBH. Interestingly, the loop deletions increased activity on amorphous cellulose, which illustrates the importance of the choice of substrate when evaluating enzyme performance. The abovementioned positive mutational effects on the efficiency of CBHs have been ascribed to increased dissociation rates, which, next to speeding up the catalytic cycle, may also affect the ability of the enzyme to dissociate when encountering an obstacle (Kurašin and Väljamäe, 2011). Importantly, mutational effects will be substrate-dependent, since the rate-limiting step in catalysis will vary between hard-to-access crystalline substrates, where association-promoting strong affinity may be beneficial, and more easily accessible, less crystalline (amorphous) substrates, where the dissociation step may be rate-limiting, as beautifully shown in early work on chitinases (Horn *et al.*, 2006, Zakariassen *et al.*, 2010).

Engineering studies demonstrating improved degradation of (heterogeneous) pretreated lignocellulolytic substrates, which is a more relevant success parameter from an industrial viewpoint, are rare. One key example is an engineered variant of *Tr*Cel7A that was generated based on the structure of a natural GH7 from *Penicillium funiculosum* (*Pf*Cel7A) showing enhanced activity on pretreated corn stover (when using *Pf*Cel7A, the time needed to reach 80% conversion of the cellulose was reduced by 60%). Inspired by *Pf*Cel7A, Taylor *et al.* (2018) removed a disulfide bond and shortened a substrate-enclosing loop near the tunnel entrance in *Tr*Cel7A, which yielded a more efficient variant of this enzyme, reducing the time needed to reach 80% conversion by approximately 30% (Taylor *et al.*, 2018).

Variation in substrate affinity may also be achieved by engineering existing CBMs or by deleting or appending CBMs. This is a popular engineering strategy, the value of which for industrial biomass processing remains somewhat unclear (Cruys-Bagger *et al.*, 2013, Várnai *et al.*, 2013), which is discussed below. Importantly, the impact of CBMs on the potentially rate-limiting enzyme properties discussed above, i.e. substrate and product affinity as well as processive movement, is not very clear and may be limited (Bommarius *et al.*, 2014).

#### Endoglucanases (EGs)

As alluded to above, endoglucanases occur in many GH families. The most studied endoglucanases belong to the large, functionally heterogeneous and widely spread GH5 family, and to family GH7. *Tr*Cel7B, also known as *Tr*EGI, is a well-known endoglucanase (Payne *et al.*, 2015) with a documented impact on the overall efficiency of cellulolytic enzyme cocktails from *T. reesei* (Chylenski *et al.*, 2017). Aiming primarily at improved stability and activity at higher temperatures, Chokhawala *et al.* (2015) used site saturation mutagenesis at seven target sites to generate *Tr*Cel7B variants. One of the selected variants (G230A/D113S/D115T) had 2-fold improved activity at 65°C on Avicel and its thermostability was also improved significantly. Using saturation mutagenesis of a single residue in a substrate-binding loop in a GH5 from *Gloeophyllum trabeum*, Zheng *et al.* (2018) were able to increase the activity of this enzyme on barley β-glucan by 1.3 to 1.5-fold. In another study, error-prone PCR-mediated directed evolution of an endo-β-1,4-glucanase from *Streptomyces* sp. G12 led to a 30% improvement in bioconversion yields for pretreated *Arundo donax* biomass (Cecchini *et al.*, 2018). Chen *et al.* (2018) engineered a β-1,4-endoglucanase from *Chaetomium thermophilum* through site-directed mutagenesis of noncatalytic residues involved in substrate binding. Two single mutations, Y30F and Y173F, increased the enzyme’s specific activity toward using carboxymethylcellulose sodium (CMC-Na) by 1.4- and 1.9-fold, respectively. Torktaz *et al.* (2018) engineered Cel5E from *Clostridium thermocellum* by rational mutagenesis and found that individual mutations N94W, N94F, E133F and N94A improved activity on carboxymethyl cellulose (CMC) and barley β-glucan by 1.1- to 1.9-fold. As a final example, Aich and Datta (2020) reported a 2-fold increase in catalytic activity on CMC by engineering conserved residues in the substrate-binding tunnel and on the surface of a thermostable GH7 endoglucanase from *Bipolaris sorokiniana*.

#### β-Glucosidases (BGLs)

While not being true cellulases, it is important to mention β-glucosidases, which are needed to complete cellulose saccharification. The dual purpose of BGLs in total saccharification of cellulose is to produce free glucose and to alleviate end-product inhibition of CBHs and EGs during saccharification. Notably, BGL itself is also prone to end-product inhibition, by glucose. Due to their important role and identified limitations, BGLs have been the subject of many engineering studies addressing catalytic activity, stability, pH-activity profile, and substrate and product inhibition (Supplementary Table 1).

Recent examples, all addressing fungal enzymes belonging to families GH1 or GH3, include the following: (i) site saturation mutagenesis of amino acids forming the catalytic pocket to increase activity toward cellobiose (Baba *et al.*, 2016); (ii) rational site-directed mutagenesis of active site residues to improve glucose tolerance (Santos *et al.*, 2019); and (iii) directed evolution (error-prone PCR + screening) to simultaneously increase the *k*_cat_/*K_M_* for cellobiose and reduce substrate inhibition (Kao *et al.*, 2021). Changes in catalytic activity have been achieved by various types of mutations near the catalytic site, whereas changes in the affinity for cellobiose and glucose have been achieved by removing an aromatic residue in a sugar-binding subsite (F256M; Kao *et al.*, 2021) and by narrowing the entrance to the substrate-binding pocket (L167W + P172L; Santos *et al.*, 2019), respectively.

### Engineering for enzyme stability

#### Thermal stability

Low thermal stability and rapid loss of catalytic performance of key cellulase components at industrially required, or desirable, temperatures is one of the main concerns in cellulase cocktail development. Hence, the literature is rich in studies showing the engineering of individual cellulases with increased thermal stability, using both rational and random approaches that are well-known from studies on other enzymes (Eijsink *et al.*, 2004, Eijsink *et al.*, 2005, Liu *et al.*, 2019, Patel *et al.*, 2019, Planas-Iglesias *et al.*, 2021). Engineering stability is rather feasible because, although the structural features governing enzyme activity vary from enzyme to enzyme, structural features governing enzyme stability are more, albeit far from fully, universal (see Eijsink *et al.*, 2004, for further discussion). In addition, high-throughput screening of enzyme variants is relatively easy when assessing stability, since one can use chromogenic, substrates compared to screening for, for example, catalytic activity on pretreated lignocellulosic biomass. It is important to note that efficient industrial processing of lignocellulosic biomass requires a complex enzyme blend. Thus, although improving the stability of an individual member of the blend may have beneficial effects on process costs, this will not normally be sufficient to allow running the process at higher temperatures as the stability of other enzymes will become limited.

One approach for cellulase stabilization has been to insert disulfide bridges, as shown by e.g. Voutilainen *et al.* (2010) and illustrated by a recent study of Bashirova *et al.* (2019), who generated two enzyme variants of a GH5 EG from *Penicilllium verruculosum* by introducing a disulfide bridge through mutations S127C-A165C or Y171C-L201C. Both variants displayed increased specific activity toward carboxymethylcellulose and barley β-glucan at 50 °C as well as increased thermal stability. Introducing disulfide bridges is just one of several more or less ‘general’ strategies for rational engineering of enzyme stability. Importantly, also free cysteines need attention since they may have a negative effect on thermal stability due to their sensitivity for oxidative damage and/or ability to form intermolecular disulfide bridges. For example, Wu *et al.* (2013) showed that removal of a free cysteine in Cel6A from *H. jecorina* resulted in increased thermal stability, whereas introduction of a free cysteine led to reduced stability. Similar trends were observed by Yamaguchi *et al.* (2020) when engineering the thermal stability of *Pc*Cel6A from *Phanerochaete chrysosporium*.

The SCHEMA technology developed by Frances Arnold and her team uses a computational algorithm to identify fragments of proteins that can be recombined without disturbing the integrity of the three-dimensional structure (Voigt *et al.*, 2002). The use of this directed evolution technology for generating cellulases with increased stability has been highly successful. For example, Heinzelman *et al.* (2009) used this technology to recombine three fungal CBH IIs, yielding 6561 possible chimeric sequences. Screening of this rather limited library yielded multiple enzymes with drastically increased thermal stability and increased activity at elevated temperatures. For some mutants, the half-life at 63°C was increased by one to two orders of magnitude.

**Fig. 4:**
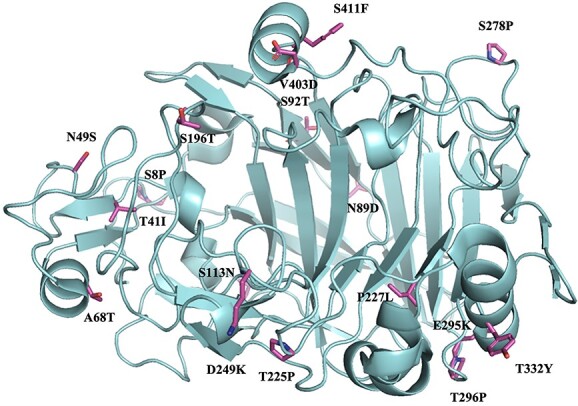
Crystal structure of the catalytic domain of an engineered thermostable *Tr*Cel7A variant with 17 mutations. The side chains of the inserted residues are highlighted in stick representation, (PDB code 5OA5) (Goedegebuur *et al.*, 2017). This thermostable variant was obtained through directed evolution and contained one more mutation at the linker-CBM junction. The stable variant exhibited a 10.4°C increase in *T*_m_ and a 44-fold greater half-life at 62 °C, compared with the wild-type enzyme. The side chains of the residues inserted into the stable mutant are shown in magenta and labeled by the mutation. The graph shows that the mutations are distributed over a large area and are mainly located on the surface of the protein, except for the P227L mutation where the side chain is completely buried within the protein. The four introduced prolines occur in surface loops.

As another example, Goedegebuur *et al.* (2017) obtained a 10.4°C increase in the apparent melting temperature and a 44-fold increase in half-life at 62°C upon directed evolution of *Tr*Cel7A from *T. reesei* (Fig. 4). Yoav *et al.* (2019) obtained a thermostable mutant with a 6.4°C increase in inflection temperature (T^i^) upon random mutagenesis of β-glucosidase from *C. thermocellum*. Zheng *et al.* (2019) also obtained highly thermostable GH5 variants through the production of chimeras of Egl5A from *T. emersonii* (*Te*Egl5A) with Cel5 from *S. opalus* (*So*Cel5). As another example, using an innovative directed evolution approach, Contreras *et al.* (2020b) obtained a 7.7°C increase in melting temperature of the endo-β-1,4-glucanase *Pv*Cel5A from *P. verruculosum* and a 5.5-fold greater half-life at 75 °C.

Of note, the stability of cellulases may be affected by the presence of additional domains (CBMs) and linkers, as well as by the degree of glycosylation of these domains and linkers (Amore *et al.*, 2017). This provides additional targets for cellulase engineering.

#### Engineering pH-dependent activity and stability

The pH of enzymatic processes is normally adjusted to the pH optima of the enzymes. For instance, commercial saccharification of lignocellulosic biomass into fermentable sugars is typically carried out with a fungal cellulase cocktail at a mildly acidic pH, which is the optimal pH of fungal growth and indeed of most fungal cellulases. To avoid enzyme inactivation due to fluctuations in pH, fungal cellulases may be engineered for robustness, i.e. stability at a broader pH range. As an example, Xia *et al.* (2016) found that removing potential O-glycosylation sites by site-directed mutagenesis broadened the operational pH range of a GH3 BGL, *Tl*BGL3A from *Talaromyces leycettanus*, from 4.0–5.0 to 3.0–10.0. Cellulases with extreme pH optimum for special applications are most often sought after and obtained through bioprospecting, capitalizing on the rapidly increasing number of extremophiles with sequenced genomes (Ben Hmad and Gargouri, 2017).

#### Engineering cellulase modularity

Cellulolytic enzymes (CBHs, EGs and LPMOs) catalyze depolymerization of insoluble cellulose at the solid–liquid interface. The catalytic domains (CD) of these enzymes often carry a CBM that improves substrate-binding and may target the CD to specific areas of the substrate (Boraston *et al.*, 2004). Some bacterial cellulases are truly multimodular and multifunctional, comprising multiple CBMs and two or more CDs with complementary activities, which degrade the substrate synergistically (Brunecky *et al.*, 2017). Accordingly, options to improve cellulase characteristics include the generation of chimeric multi-domain proteins by fusing one or more CBMs to a single-domain cellulase, by replacing the CBM of multi-domain cellulases with alternate CBMs, or by fusing CDs with different activities. The problem with these potential approaches is that they are difficult to rationalize. As just one example, CBM effects vary between cellulases and between substrates (e.g. Duan *et al.*, 2017). As to CBMs, an additional issue is that the impact of the domains varies with substrate availability: at high substrate concentrations, e.g. in the beginning of an industrial batch-wise bioprocess, CBMs are not needed and may even slow down cellulases while, on the other hand, CBMs promote cellulase efficiency at lower substrate concentrations, e.g., in the later phases of the bioprocess (Le Costaouëc *et al.*, 2013, Várnai *et al.*, 2013, Badino *et al.*, 2017, Jensen *et al.*, 2018, Christensen *et al.*, 2019).

The most common cellulose specific CBMs belong to family CBM1, exclusively found in fungi, and family CBM2, found in bacteria (Sammond *et al.*, 2012). CBMs may be appended to both the C- or N-terminus of GHs, and their position may be characteristic for certain GH families. For example, CBM1s tend to be appended to the C-terminus in fungal GH7 CBHs and EGs and to the N-terminus in fungal GH6 CBHs and GH5 EGs. Several studies have shown that fusing an additional or alternate CBM to the CD of a cellulase may improve catalytic efficiency toward insoluble cellulosic substrates and/or thermostability (Voutilainen *et al.*, 2014, Oliveira *et al.*, 2015, Sadat, 2017, Christensen *et al.*, 2019, Gilmore *et al.*, 2020). An early study led to the conclusion that the addition of a CBM is more beneficial for EGs compared with CBHs (Szijártó *et al.*, 2008). It must be emphasized that naturally occurring CBMs show different cellulose-binding features (Carrard *et al.*, 2000, Lehtiö *et al.*, 2003) and that such features also may be changed through protein engineering (see, e.g. Takashima *et al.*, 2007, Chen *et al.*, 2014).

In nature, CBMs and CDs are coupled by linkers of varying lengths and sequences. For example, linkers in fungal GH6/GH7 enzymes are rich in serine and threonine, whereas bacterial cellulase linkers have higher proline content (Sammond *et al.*, 2012, Payne *et al.*, 2015). This difference may have to do with protection against proteolytic attack, which for fungal linkers is prevented by glycosylation (Fig. 2), whereas bacteria, with their limited protein glycosylation abilities, need to incorporate proline residues. There are indications that the position (N- vs. C-terminal) as well as the length and amino acid sequence of linkers have co-evolved with the domains that they connect for optimal function (Sammond *et al.*, 2012, Payne *et al.*, 2015). In general, the amino acid composition determines flexibility, while linker length affects the distance between the connected domains. Importantly, both computational (Payne *et al.*, 2013) and experimental (Chen *et al.*, 2014) work has shown that the glycosylations on the linkers and CBMs of fungal cellulases have multiple effects on enzyme function. Using molecular dynamics (MD) simulations, Payne *et al.* (2013) showed that the glycosyl moieties of linkers in *T. reesei* GH6 (*Tr*Cel6A) and GH7 CBHs (*Tr*Cel7A) make considerable contributions to substrate affinity. Chen *et al.* (2014) investigated the effects of *O*-mannosylation on the CBM1 of *T. reesei* GH7 CBHs (*Tr*Cel7A) and found that glycosylation promoted both enzyme stability and substrate affinity. Despite growing awareness of the impact of linker types and length on the catalytic activity of cellulases [e.g. on processivity (Wang *et al.*, 2018) or substrate affinity (Nakamura *et al.*, 2016)], little is known about how this knowledge may be applied to design or engineer linkers for fusion proteins.

## Engineering cellulase cocktails

Commercial cellulase products that are used for saccharification of lignocellulosic feedstocks are the result of decades of extensive strain development to generate production organisms that secrete an optimized blend of cellulases in large amounts, at low cost. In general, cellulase cocktails are fungal secretomes and can thus be improved indirectly by supplementing with another enzyme cocktail or individual enzymes, or directly by genetic modification of the production strain. Cellulase production strains are engineered to modulate transcription and secretion of cellulases, to remove undesirable background activities (e.g., proteases), to replace selected enzyme components of the cellulolytic blend with more potent, naturally occurring or engineered variants, or by inserting (i.e. knocking in) genes encoding additional enzymes. The composition of the resulting cellulase cocktails, i.e., the engineered fungal secretomes, may be optimized further by varying process conditions (including the carbon source) during cellulase expression. Approaches to improve cellulase cocktails have been reviewed recently in detail by Østby et al. (2020).

Perhaps the most remarkable (well-documented) example of strain engineering is the development of the RUT-C30 cellulase hyperproducer prototype strain, which is derived from strain QM6a, and shows a 20-fold increase in extracellular protein levels, reaching about 30 g/L on a lactose-containing growth medium (Bischof *et al.*, 2016). Recent tools such as RNAi-mediated gene silencing (Sun *et al.*, 2022), inducer-free expression systems (Arai *et al.*, 2022), CRISPR/Cas9-based methods (Rantasalo *et al.*, 2019, Fonseca *et al.*, 2020) and synthetic expression systems with engineered transcription factors and promoters (Rantasalo *et al.*, 2018) provide new tools for engineering cellulase production strains (Mojzita *et al.*, 2019). For example, Arai *et al.* (2022) reported an inducer-free expression system for *T. reesei* that enables protein overproduction in glucose– containing media without inducers like cellulose, lactose and sophorose. In another study, Fonseca *et al.* (2020) used CRISPR/Cas9-based rational engineering of the RUT-C30 strain to increase protein secretion and β-glucosidase (72-fold) and xylanase (42-fold) activities in the secretome. Implemented changes included the constitutive expression of a mutant of the cellulase master regulator XYR1, heterologous expression of the *Te*Cel3A BGL from *T. emersonii,* and deletion of genes encoding for the cellulase repressor ACE1 and the proteases SLP1 and PEP1. By combining CRISPR/Cas9 technologies with an exquisite genetic toolbox, Rantasalo *et al.* (2019) have developed versatile *T. reesei*-based expression systems that allow rapid construction of recombinant strains that produce high levels of secreted protein using glucose as a carbon source.

## Concluding remarks

Rational protein engineering and directed evolution have contributed to our current understanding of enzymatic cellulose saccharification and to the efficiency of current commercial cellulolytic enzyme cocktails. As to the latter, it must be noted that the work described above and in other recent reviews (Payne *et al.*, 2015, Contreras *et al.*, 2020a, Zhang *et al.*, 2021) likely only shows the tip of an iceberg of cellulase engineering, since work done by commercial enzyme suppliers is largely unknown to the scientific community. When assessing publicly available data on cellulases and cellulase engineering, it is important to note that quite some work is based on the use of cellulose substrates that are relatively easy to degrade and of little relevance to the biorefinery, such as carboxymethylcellulose. It needs to be emphasized once more that, when working toward industrial applications, characterization of wild-type cellulases and of mutational effects needs to be done with relevant substrates using relevant (process) conditions.

A particular challenge in cellulase engineering lies in the complex interplay between different enzyme traits, which complicates the design of enzymes that are ‘generally efficient’. For example, product inhibition in one of the key fungal cellulases, *Tr*Cel7A, is a result of strong interactions in the product binding site that, at the same time, are vital for the processive mechanism that is necessary for being able to degrade the more crystalline parts of cellulose (Kuusk *et al.*, 2015, Olsen *et al.*, 2020). It is possible to reduce product inhibition by reducing strong binding interactions in product sites through site-directed mutagenesis, but this comes at a cost of lower enzyme efficiency when working with crystalline substrates (Atreya *et al.*, 2016, Olsen *et al.*, 2020). To make things even more complicated, the need for processivity may vary as a saccharification reaction proceeds and the concentration and composition of the substrate change. It is also worth noting that LPMO action on the crystalline surfaces of cellulose may increase accessibility to cellulases that are less capable of acting on a crystalline material, but intrinsically faster (Hamre *et al.*, 2019).

Of course, different substrates, for example varying in cellulose crystallinity, will require different cellulase blends containing enzymes with different properties, and it is now well established that the development of enzyme technology for the future lignocellulosic biorefinery cannot be based on a one-size-fits-all approach (Banerjee *et al.*, 2010, Hu *et al.*, 2014, Du *et al.*, 2020, Østby *et al.*, 2020). As alluded to several times above, it is a long way from engineering a single cellulase with improved properties to producing improved enzyme blends for various common lignocellulosic substrates such as pretreated corn stover or steam-exploded wood chips.

Saccharification of lignocellulosic biomass, and even pure cellulose, requires multiple enzymes, which means that engineered improved variants of single cellulases are not useful if their synergistic action with other enzymes is not maintained. The synergy between various cellulase types is a topic of great interest and debate (e.g. Igarashi *et al.*, 2011, Jalak *et al.*, 2012, Zajki-Zechmeister *et al.*, 2022) that we did not address in the above, but that needs ample attention when developing cellulase cocktails rather than individual enzymes.

Despite major progress in the past decades, further improvements in cellulase efficiency still seem feasible, targeting the properties discussed above and exploiting the latest developments in computational tools for protein engineering. Alphafold (Jumper *et al.*, 2021) provides rapid access to structural evaluation of natural diversity, expanding the knowledge base for protein engineering. Machine learning tools enable rapid extraction of functionally crucial enzyme features from sequences and structures. For example, using machine-learning models trained only on the number of residues in the active-site loops, Gado *et al.* (2021) were able to predict the CBH or EG nature of GH7 family members, as well as the presence of a CBM, with high accuracy. Their models not only provide plausible explanations for the results of several engineering studies already in the literature (and reviewed here), but also point at novel engineering targets for GH7s.

An important development for cellulase engineering comes from the work of Peter Westh and colleagues, who have developed a new framework for describing, interpreting and predicting the kinetics of heterogeneous enzyme catalysis, i.e. enzyme catalysis at a solid–liquid interface, as is the case for cellulases (Kari *et al.*, 2018, Schaller *et al.*, 2021, Kari *et al.*, 2022). One key finding of this work, inspired by the Sabatier principle, is the strong relationship between substrate affinity and catalytic efficiency, which had been observed before (e.g. Horn *et al.*, 2006; see above), but which, thanks to the work of Westh and colleagues now can be rationalized and quantified in a theoretical framework with predictive value. For example, Schaller *et al.*, (2021) developed a method for predicting the binding and activation free energies of cellulases that was successfully used in virtual (computational) screening of GH7 cellulases, resulting in the discovery of novel natural GH7 variants with promising catalytic performance (Schaller *et al.*, 2022). As another example, Kari *et al.* (2021) produced and kinetically characterized 83 cellulases, which allowed them to reveal a linear free energy relationship between the substrate binding strength and the activation barrier, thus underpinning the predictive power of calculated substrate binding strengths.

From the perspective of industrial applications, it would be wise to base future efforts on a deeper understanding of the interplay between the various members of cellulolytic enzyme cocktails. In this respect, the interplay between cellulases and the relatively recently discovered LPMOs deserves special attention. LPMOs not only add an endo-type of chain cleaving activity to the cocktail, they also generate a new type of chain ends carrying oxidations. It has already been demonstrated that LPMO action changes the importance of processive action (Hamre *et al.*, 2019) and that the synergy between LPMOs and cellulases depends on both the substrate and the cellulase (Tokin *et al.*, 2020). Engineering CBHs to better interact with such oxidized chain ends could be one way to improve synergistic effects between cellulases and LPMOs. Of note, the LPMOs themselves are of course also interesting targets for protein engineering, addressing, for example, their stability under turnover conditions (Forsberg *et al.*, 2020). Simultaneous focus on a deeper understanding and better exploitation of LPMO and cellulase action may reveal new engineering targets and lead to further improvement of current cellulolytic enzyme cocktails.

## Supplementary Material

PEDS22_0053_R1_Supplementary_material_Clean_gzad002

## Data Availability

No new data were generated in this manuscript.

## Notes

Edited by: Dr Eva Petersen
