# Supplementary material for: Engineering cellulases for conversion of lignocellulosic biomass
Source: Protein Eng Des Sel. 2023 Mar 24;36:gzad002. doi: 10.1093/protein/gzad002 (PMC10394125; doi:10.1093/protein/gzad002)
Supplement: PEDS22_0053_R1_Supplementary_material_Clean_gzad002 [file peds22_0053_r1_supplementary_material_clean_gzad002.docx]

**Supplementary material**

**Engineering cellulases for conversion of lignocellulosic biomass**

Yogesh Babasaheb Chaudhari^1^, Anikó Várnai^1^, Morten Sørlie^1^, Svein Jarle Horn^1^, Vincent G. H. Eijsink^1^

^1^Faculty of Chemistry, Biotechnology, and Food Science, NMBU-Norwegian University of Life Sciences, P.O. Box 5003, 1432, Ås, Norway

**Email address:**

yogesh.chaudhari@nmbu.no

aniko.varnai@nmbu.no

morten.sorlie@nmbu.no

svein.horn@nmbu.no

vincent.eijsink@nmbu.no

**Supplementary Table 1. Examples of protein engineering studies with cellulases.** This Table focuses on recent work, while including a few older examples. See Contreras *et al.* (2020), Zhang *et al.* (2021) for similar Tables.

| **GH Family** | **Protein** | **Expression host** | **Engineering approach** | **Comments** | **References** |
| --- | --- | --- | --- | --- | --- |
| GH5 | GH5 cellulase from *Gloeophyllum trabeum* CBS 900.73 | *P. pastoris* GS115 | Site saturation mutagenesis of position 233 in a surface loop with substrate interactions. | Specific activity on barley β-glucan increased by 1.3 to 1.5-fold. | Zheng *et al.*, 2018 |
| GH5 | CelH from *Clostridium thermocellum* | *E. coli* BL21 (DE3) | Site-directed mutagenesis of amino acids near the Cel5E active site. | Increased activity on carboxymethyl cellulose and β-glucan by 1.9- and 1.4-fold, respectively. | Torktaz *et al.*, 2018 |
| GH5 | EGLII from *P. verruculosum* | *P. canescens* PCA10 strain | Insertion of disulfide bonds. | Improved activity (15-22%) on carboxymethyl-cellulose and β-glucan. Improved stability at 70 and 80°C. | Bashirova *et al.*, 2019 |
| GH5 | Egl5A from *Talaromyces emersonii* and Cel5 from *Stegonsporium opalus* | *P. pastoris* GS115 | Structure-guided fusion of Egl5A from *T. emersonii* (*Te*Egl5A) with Cel5 from *S. opalus* (*So*Cel5). | Up to 19 °C increase in T_50_, 22.9°C increase in T_m_ and 650-fold increase inb t_1/2_ at 55°C, relative to the mesophilic parent. | Zheng *et al.*, 2019 |
| GH5 | Cel5A from *Penicillium verruculosum* | *P. pastoris* BSYBG11 | KnowVolution (knowledge-gaining directed evolution strategy)-site saturation mutagenesis and recombination, ~1980 variants screened for thermal stability at 75 °C. | Increase in t_1/2_ at 75 °C by 5.5-fold_,_ (32 to 175 min); ΔT_m_ = + 7.7 °C. | Contreras *et al.*, 2020 |
| GH6 | Cel6A from *H. jecorina, Humicola insolens* and *Chaetomium thermophilum* | *S. cerevisiae* ATCC No. 4014317 | SCHEMA structure-guided recombination of 3 fungal CBHII (6561 variants screened). | > 1 order of magnitude increase in t_1/2_ at 63 ^o^C. | Heinzelman *et al.*, 2009 |
| GH6 | 3C6P an engineered variant of Cel6A from *H. jecorina* | *S. cerevisiae* ATCC No. 4014317 | Removal of a free cysteines. | Increase in half-life at 90 ^o^C from less than 5 min to 76 min (at pH 6.0) | Wu *et al.*, 2013 |
| GH6 | Cel6A from *Phanerochaete chrysosporium* | *P. pastoris* KM71H | Random and site-directed mutagenesis, including mutation of free cysteines. | Improved enzyme performance 60 °C | Yamaguchi *et al.*, 2020 |
| GH7 | Cel7A from a *Talaromyces emersonii* | *S. cerevisiae* | Structure-guided disulfide engineering; addition of disulfide bridges in or between loops shaping the active site tunnel. | Improved thermostability to T_m_ = 84°C (ΔT_m_ = +9°C); >10- fold increase half-life time at 70 ^o^C (t_1/2_ = 320 min); increased activity on Avicel at higher temperatures. | Voutilainen *et al.*, 2010 |
| GH7 | Cel7A from *T. reesei* | *T. reesei*ALKO 3413 | Replacement of a substrate-binding tryptophane in the -7 subsite (the “entrance” of the substrate-binding tunnel). | Reduced activity of crystalline cellulose; increased activity on amorphous cellulose. | Nakamura *et al.*, 2013 |
| GH7 | Cel7A from *T. reesei* | A. oryzae | Replacement of a substrate-binding tryptophane in the -4 subsite. | Lower substrate affinity but two-fold higher activity on Avicel. | Kari *et al.*, 2014 |
| GH7 | Cel7B from *T. reesei* | *T. reesei* QM9414 | Site saturation mutagenesis at 7 sites targeting one – three codons at each site; screening of 11,000 mutants. | Two-fold improved activity at 65°C on Avicel and two-fold longer half-life at 60°C. | Chokhawala *et al.*, 2015 |
| GH7 | Cel 7A from *Rasamsonia emersonii* | *A. oryzae* | Site-directed mutations in a substrate-enclosing loop (-4 subsite) that weaken substrate affinity (N194A/N197A). | Reduced substrate affinity (likely increased dissociation) and two-fold higher activity on Avicel. | Sørensen *et al.*, 2017 |
| GH7 | Cel7A from *T. reesei* | *A. niger var. awamori* AP4 | Recombination of pre-selected stable single-point mutants (~ 2000 screened). | Increased T_m_ by up to 10.4 °C; up to 9-fold increase t_1/2_ at 69 °C. | Goedegebuur *et al.*, 2017 |
| GH7 | Cel7A from *T. reesei* | *T. reesei* | Removal of a disulfide bridge and shortening of a substrate-enclosing loop. | Time needed to reach 80 % conversion of pre-treated corn stover reduced by some 30%. | Taylor *et al.*, 2018 |
| GH7 | Cel7A from *T. reesei* | *Aspergillus oryzae* | Rational loop engineering to change exo/endo behaviour. | Identification of a substrate-enclosing loop that determines exo-/endo-behaviour; mutants with up higher activity on amorphous cellulose and lower activity on crystalline cellulose. | Schiano-di-Cola *et al.*, 2019 |
| GH7 | Endoglucanase from *Bipolaris sorokiniana* | *P. pastoris* X33 | Site-directed mutagenesis of amino acids in the substrate binding tunnel (D257W and Q225H) and on the protein surface (Y222R and Q401N). | Increased specific activity on carboxymethylcellulose by 2-fold. | Aich and Datta, 2020 |
| GH12 | Endo-β-1,4-glucanase from *Streptomyces* sp. G12 | *E. coli* | Error-prone PCR, with ~2200 variants screened. | 30% enhanced saccharification of pretreated *Arundo donax.* | Cecchini *et al.*, 2018 |
| GH45 | *Chaetomium thermophilum* β-1,4-endoglucanase | *P. pastoris* | Site saturation mutagenesis of noncatalytic residues located around the substrate binding site. | Increased specific activity on carboxymethylcellulose by 1.9-fold. | Chen *et al.*, 2018 |
| **β-glucosidases** | | | | | |
| GH1 | Bgl from *T. harzianum* | *E. coli* Rosetta | Site-directed mutagenesis of the amino acids involved in substrate/product binding. | Increased activity (3-5 fold) on p-nitrophenyl-β-glucopyranoside and increased glucose tolerance. | Santos *et al.*, 2019 |
| GH1 | BGL from *Clostridium thermocellum* | *E. coli* BL21DE3 | Random mutagenesis (~8000 variants screened), one potential variant (A17) obtained. | Increase in the T giving 50 % inactivation after 1 h incubation by 6.4 °C | Yoav *et al.*, 2019 |
| GH3 | Bgl3A from *Talaromyces leycettanus* JCM12802 | *P. pastoris* GS115 | Removal of O-glycosylation sites by site-directed mutagenesis. | Broadened operational pH range, from 4.0–5.0 to 3.0–10.0. | Xia *et al.*, 2016 |
| GH3 | BGL1 from *Aspergillus aculeatus* | *Saccharomyces cerevisiae* DC5 | Site saturation mutagenesis in the catalytic pocket, with ~4860 variants screened. | Enhanced *k_c_*_at_/*K*_m_ for cellobiose by 2.7-fold. | Baba *et al.*, 2016 |
| GH3 | D2‑BGL from *Chaetomella raphigera* | *P. pastoris* | Site‑directed mutagenesis, (~27000 variants screened). | 1.3 times higher *V*_max_ and *K*_m_ values for cellobiose; improved tolerance to substrate inhibition. | Kao *et al.*, 2021 |
| **CBM/linker engineering** | | | | | |
| GH7 and GH45 | Cel7A, Cel7B and Cel45A from *Melanocarpus albomyces* | *T. reesei* (A33) | Removing and adding CBMs. | The presence of a CBM improves hydrolysis of various cellulosic substrates. | Szijártó *et al.*, 2008 |
| GH7 | CBM free Cel7A from *T. aurantiacus* | *T. reesei* | C-terminal fusion of CBM from *C. thermophilum* Cel7A and *T. reesei* Cel7A. | Enhanced hydrolysis of Avicel by 2 to 4-fold at 45 °C and at 70 °C. | Voutilainen *et al.*, 2008 |
| GH7 | CBM-free Cel7A from *Talaromyces emersonii (Te*Cel7A*)* | *S. cerevisiae* | Adding a CBM1, CBM2 or CBM3. | Better binding to Avicel and up to 6-fold increased activity. | Voutilainen *et al.*, 2014 |
| GH7 | The CBM1 of Cel7A from *T. reesei* (*Tr*Cel7A) | Peptide synthesis | Manipulating the glycosylation state of the CBM. | The glycosylation state of the CBM affects thermal stability and the affinity for cellulose. | Chen *et al.*, 2014 |
| GH9 | A CBM-free endoglucanase (UmCel9A) from an uncultured microorganism | *E. coli* Rosetta (DE3) pLysS | C-terminal addition of 5 CBMs (CBM 1, 2, 3, 10 and 72) or N-terminal of a CBM4 using native linkers. | Enhanced activity on PASC, alkali-pretreated sugarcane bagasse, filter paper powder and Avicel by 4.2-, 3.0-, 2.4, and 6.6-fold, respectively. | Duan *et al.*, 2017 |
| GH7 | Cel7A from *T. reesei* (*Tr*Cel7A) | *A. oryzae* | Linker engineering [deletion of 6 residues close to catalytic domain (variant 1), substitution of 4 *O*-glycosylated residues in the middle of the linker (variant 2), and elongation of the linker near the CBM (variant 3). | Variants 2 & 3 reduce affinity and increase activity for Avicel. Interesting insights in CBM function. | Badino *et al.*, 2017 |
| GH5 | CBM-containing Endoglucanase I from *Volvariella volvacea* | *P. pastoris* strain KM71H | Eight EG1 variants with varying linker length, flexibility, and/or sequence. | The linker impacts enzyme performance; the best variant released about 30 % more sugar during hydrolysis of filter paper | Wang *et al.*, 2018 |
| GH6 | Cellulase from metagenome of a thermophilic microbial community (mgCel6A) | *E. coli* BL21DE3 | Deletion of CBM2 domain from mgCel6A. | Reduction in substrate affinity; reduction in activity on sulfite-pulped spruce only at lower substrate concentration. | Jensen *et al.*, 2018 |
| GH6 | Cel6A from *T. reesei* (TrC6A) | *A. oryzae* | Replace native N-terminal CBM with CBMs of *Podospora anserina*, *Neocallimastix frontalis*, *Serendipita indica*, *Coprinopsis cinerea*, and *Volvariella volvacea*. | Variations in cellulose affinity with implications for enzyme efficiency. | Christensen *et al.*, 2019 |
